# Universal free school meals and Medicaid spending: evidence from linked education and claims data in Arkansas

**DOI:** 10.1093/haschl/qxag196

**Published:** 2026-08-05

**Authors:** Joe Sundell, Harneet Kaur, Gwendolyn M Lawson, Xueying Ma, Bradley C Martin, J Mick Tilford, Joseph Thompson, Michael R Thomsen

**Affiliations:** Department of Health Policy and Management, University of Arkansas for Medical Sciences, Little Rock, AR 72205, United States; Department of Health Policy and Management, University of Arkansas for Medical Sciences, Little Rock, AR 72205, United States; Department of Child and Adolescent Psychiatry and Behavioral Sciences, Children's Hospital of Philadelphia, Philadelphia, PA 19104, United States; Department of Psychiatry, University of Pennsylvania Perelman School of Medicine, Philadelphia, PA 19104, United States; Department of Health Policy and Management, University of Arkansas for Medical Sciences, Little Rock, AR 72205, United States; Division of Pharmaceutical Evaluation & Implementation, University of Arkansas for Medical Sciences, Little Rock, AR 72205, United States; Department of Health Policy and Management, University of Arkansas for Medical Sciences, Little Rock, AR 72205, United States; Arkansas Center for Health Improvement, University of Arkansas for Medical Sciences, Little Rock, AR 72201, United States; Department of Health Policy and Management, University of Arkansas for Medical Sciences, Little Rock, AR 72205, United States

**Keywords:** universal free school meals, healthcare expenditures, claims data, child nutrition policy, Medicaid costs

## Abstract

**Introduction:**

Universal free meal (UFM) policies provide school breakfast and lunch at no cost to all students, increasing participation, improving dietary quality, and reducing household food insecurity. Whether these policies affect children's health care spending is unknown.

**Methods:**

Using linked Arkansas education and Medicaid claims data (2013-2020) and a stacked difference-in-differences design across 4 UFM adoption cohorts, we examined changes in Medicaid spending among 131 851 continuously enrolled Medicaid children before and after school-level UFM adoption.

**Results:**

The aggregate effect across the 2 post-adoption years was not statistically significant (spending ratio 0.973; 95% CI 0.932-1.016). However, spending declined significantly in the second post-adoption year (spending ratio 0.937; 95% CI 0.889-0.987), a 6.3% reduction. Reductions were concentrated in inpatient and pharmacy spending for mental, behavioral, and neurodevelopmental disorders, alongside increased outpatient spending in these categories.

**Conclusions:**

UFM adoption was associated with a delayed but significant reduction in Medicaid spending, driven largely by shifts in mental and behavioral health care utilization. These findings suggest school nutrition policy may influence health care spending among publicly insured children.

## Introduction

Universal free meals (UFM) is a school nutrition policy that provides free breakfast and lunch to all students, regardless of income. By removing eligibility and paperwork barriers, UFM increases student participation in school meals, improves dietary quality, and reduces stigma associated with means-tested programs.^1,2^ Adoption of UFM has expanded across the United States, in large part through the community eligibility provision (CEP). CEP allows schools or districts with a threshold share of students participating in means-tested programs to provide meals free to all students without collecting household meal applications.^3,4^ As of the 2022-2023 school year, over 47 000 U.S. schools serving over 23 million students participated in CEP, and 8 states have enacted legislation establishing UFM statewide.^5,6^ UFM can also be implemented through Provision 2 of the National School Lunch Act, which allows free meals for up to 4 years based on reimbursement rates set in an initial base year.^7^

Research shows that UFM increases participation and improves diet quality.^8-17^ Expanded participation raises consumption of fruit, dairy, and other nutrient-dense foods.^8,18-21^ UFM also reduces household food insecurity and increases food purchasing power by eliminating school meal charges.^22-24^

A substantial body of evidence links food insecurity and poor diet quality to a range of adverse physical and mental health outcomes in children. Children in food-insecure households have higher rates of chronic disease, including asthma, attention-deficit/hyperactivity disorder (ADHD), and depression, and experience rates of foregone medical care that are 180% higher than their food-secure peers.^25-27^ The relationship is likely bidirectional in that caring for a child with chronic illness increases financial strain, while food insecurity worsens clinical conditions.^27-29^

Limited evidence indicates that interventions that reduce food insecurity can improve health outcomes and in turn reduce health expenditures. In a nationally representative analysis, Berkowitz et al. (2017) estimated that food insecurity was associated with $1863 in additional annual healthcare spending after adjusting for demographic, socioeconomic, and clinical characteristics.^30^ Related studies have shown that the Supplemental Nutrition Assistance Program (SNAP) can lower food insecurity by as much as 30%^31^ and that SNAP receipt is associated with lower annual healthcare expenditures among low-income adults, with estimated reductions of $1409^32^ and $2360^33^ per person per year in adjusted models.

Despite compelling evidence that UFM mitigates child food insecurity and improves diet quality, no study has examined whether these benefits translate into measurable differences in healthcare utilization or spending. Using a linked dataset of healthcare claims and educational records from Arkansas, we investigate whether UFM implementation in Arkansas schools between 2014 and 2020 resulted in changes to Medicaid healthcare spending among Medicaid-enrolled children using a stacked difference-in-differences (DiDs) design.

## Study data and methods

### Study design

We used a quasi-experimental DiD design to estimate changes in Medicaid expenditures associated with the adoption of UFM in Arkansas public schools. The design compares child-level changes in Medicaid spending before and after UFM exposure to contemporaneous changes among those not yet or never exposed to UFM. To account for staggered policy adoption, we implemented a stacked DiD approach that constructs balanced pre- and post-adoption event windows for each adoption cohort and pools estimates across cohorts.^34^

### Data

This study uses a linked longitudinal dataset of deidentified educational records and healthcare claims spanning September 2013-August 2020. Annual enrollment, grade level, demographic characteristics, and school identifiers were from the Arkansas Department of Education. Healthcare utilization and expenditures were obtained from the Arkansas All-Payer Claims Database (APCD), which contains enrollment and claims data for Medicaid and other insurers.^35^ These data were linked at the student level by an honest data broker at the Arkansas Center for Health Improvement. Information on school meal policy adoption was obtained from the National Center for Education Statistics using the Elementary/Secondary Information System table generator.^36^ Our exposure measure captured student attendance at a school providing UFM, regardless of whether meals were offered through CEP or Provision 2. The use of the deidentified dataset for this study was determined not to be human subjects research by the [institution omitted] Institutional Review Board (IRB #275801).

### Study subjects

**Figure 1.**
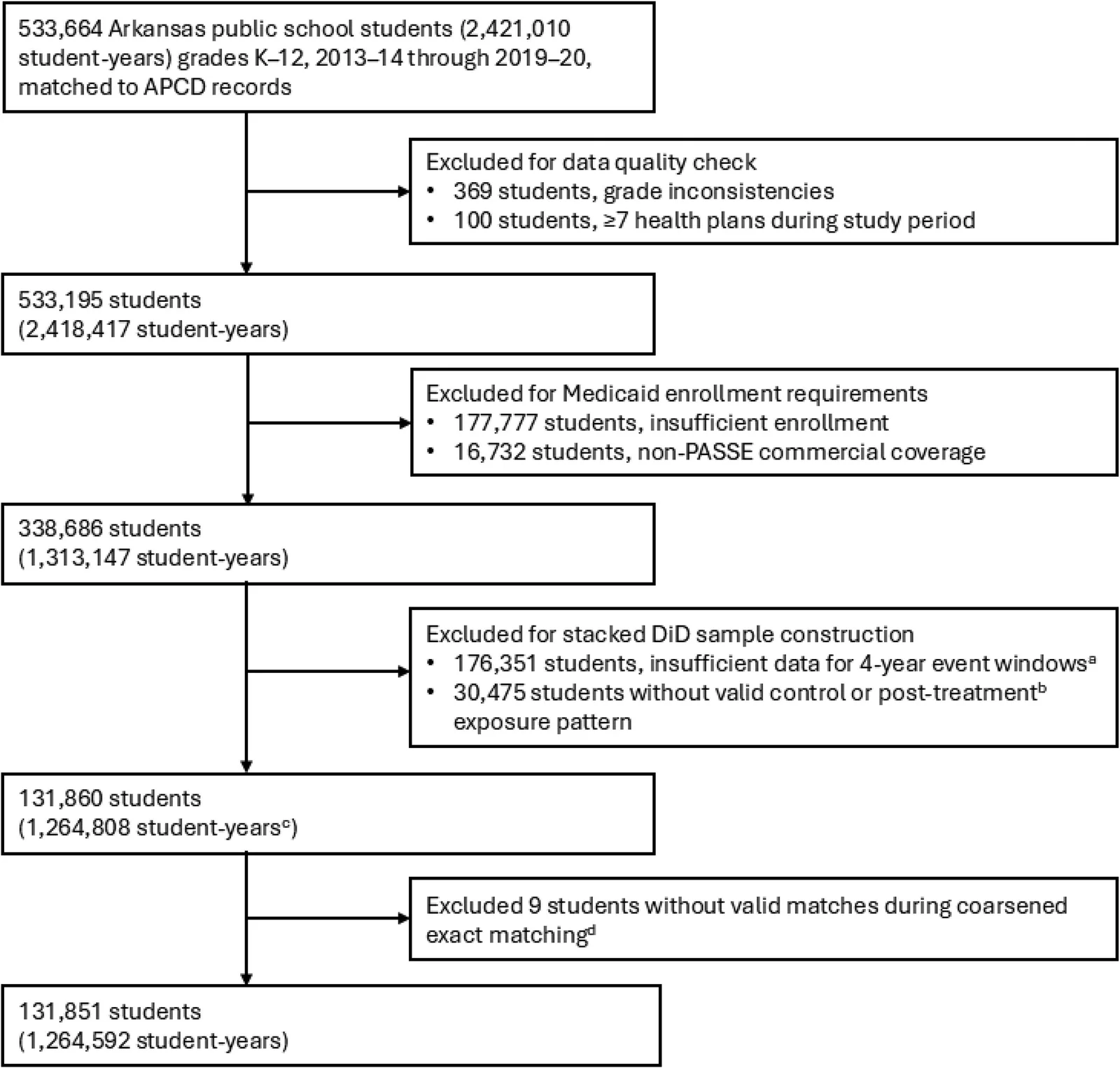
Attrition diagram for construction of the stacked difference-in-differences analytic sample. Source/Notes: SOURCE: Authors’ analysis of linked Arkansas Department of Education records and Arkansas All-Payer Claims Database, 2013-2020. NOTES: Continuous Medicaid enrollment was defined as enrollment for at least ten months between September and August of a given academic year. PASSE refers to the Provider-Led Arkansas Shared Savings Entity program. ^a^Students were excluded because they did not contribute a complete 4-year event window to any stacked difference-in-differences sub-experiment. ^b^Students contributed a complete 4-year event window to at least one sub-experiment but were excluded because their Universal Free Meals exposure pattern was inconsistent with either a clean control or treated exposure history. ^c^Student-years reflect observations contributed across sub-experiments. Because students may appear in multiple sub-experiments, the number of student-years can exceed the number of calendar-year observations in the underlying data. For example, a student observed in all study years who never received Universal Free Meals contributes 4 years of observations to each of 4 sub-experiments (16 student-years) as a clean control. A student observed from 2014 to 2019 who first receives Universal Free Meals in 2018 would contribute observations as a clean control in the 2016 sub-experiment, be excluded from the 2017 sub-experiment because the required pre/post exposure pattern is not satisfied, and contribute as a treated observation in the 2018 sub-experiment. ^d^Students were excluded after coarsened exact matching because no suitable matches were identified, and matching weights could not be assigned.

Medicaid-enrolled children attending Arkansas public school in grades K-12 from September 2013 and August 2020 were the subjects of interest. To minimize entity resolution errors, identify a stable, policy-relevant population, and ensure that observed expenditures reflected Medicaid-financed spending, we applied a series of inclusion and exclusion criteria. We first excluded students whose unique person identifier was associated with 7 or more health plans during the study period. We also excluded students with inconsistent educational trajectories, defined as a year-to-year grade change less than zero or greater than 2. We restricted the sample to student-years with continuous Medicaid enrollment, defined as at least 10 months of enrollment between a September-August year. We included students enrolled in traditional Medicaid and those covered under the Provider-Led Arkansas Shared Savings Entity (PASSE) program, a Medicaid payment model for beneficiaries with complex behavioral health or developmental conditions.^37^ Student-year observations with non-PASSE commercial coverage were excluded because additional payers may reflect different utilization and spending patterns. After applying these criteria, 338 686 students contributing 1 313 147 student-years served as the potential base population for constructing the analytic sample used in the stacked DiD analysis (Figure 1).

For sensitivity analyses, we (1) relaxed the requirement that Medicaid be the exclusive payer, allowing student-years with additional payers while keeping all other criteria unchanged and (2) re-estimated the analysis after excluding children who ever participated in the PASSE.

### Construction of the stacked difference-in-differences sample

To estimate dynamic treatment effects under staggered adoption of UFM, we followed the sub-experiment stacking approach described by Wing et al.^34^ We identified 4 cohorts of schools that adopted UFM in the 2015-2016, 2016-2017, 2017-2018,^34^ or 2018-2019 school years and, for each cohort, constructed balanced event windows spanning 2 years before and 2 years after adoption. Under the Wing et al.^34^ nomenclature, each adoption cohort constitutes a distinct sub-experiment. Because the final post-adoption year for the 2018-2019 adoption cohort overlapped with the onset of the COVID-19 pandemic, we conducted a sensitivity analysis excluding that sub-experiment to assess the robustness of findings to pandemic-related disruptions in healthcare utilization and school meal delivery.

Within each sub-experiment, we retained only students who met the continuous Medicaid enrollment requirement in all 4 academic years of the corresponding event window, ensuring that annual spending was fully observed. We further restricted the sample to students whose treatment status was consistent with either (1) no exposure to UFM within the 4 years of the sub-experiment (clean controls) or (2) no exposure during the sub-experiment pre-period followed by exposure during the sub-experiment pre-period (ie, 2 years of pretreatment observation denoted by event times −2 and −1, and 2 years of posttreatment observation denoted by event times 0 and 1). Students whose exposure patterns did not satisfy either condition were excluded from that sub-experiment.

Students could contribute observations to more than one sub-experiment if their UFM exposure pattern satisfied the inclusion criteria within multiple sub-experiments. Students who never attended a UFM school would contribute 4 years of observations to each sub-experiment as a clean control. A student observed from 2014 to 2019 who first became exposed to UFM in 2018 would contribute observations as a clean control in the 2016 sub-experiment, be excluded from the 2017 sub-experiment because the required pre/post exposure pattern is not satisfied and contribute as a treated observation in the 2018 sub-experiment. Thus, the stacked dataset does not represent a simple subset of the base population, and the number of student-year observations is not directly comparable across samples. The stacked dataset included 131 860 unique students contributing 1 264 808 student-years (Figure 1).

### Statistical analysis

The average treatment effect on the treated (ATT) was estimated using the stacked DiD estimator following Wing et al.^34^ Analytic weights were derived from coarsened exact matching, a procedure that involves coarsening continuous measures into categories and then exact matching on the coarsened data.^38^ Specifically, we defined weighting strata using sub-experiment membership and a coarsened measure of the pretreatment trend in log Medicaid spending between event times −2 and −1. Control observations were then reweighted to match the distribution of treated observations across these strata. Including sub-experiment membership in the weighting procedure ensured that treated and control observations were identically distributed across adoption cohorts, consistent with the weighting framework proposed by Wing et al.^34^ Including the coarsened pretreatment trend improved comparability between treated and control observations with respect to spending trajectories prior to UFM adoption. Observations without comparable matches were excluded, yielding a final analytic sample of 131 851 unique children contributing 1 264 592 student-year observations.

Table 1 summarizes the unweighted stacked sample, while Table A1 reports treated and control group characteristics before and after weighting. Analytic weights were applied in all regression models. Because matching on pretreatment outcomes can introduce regression-to-the-mean bias, we examined serial correlation in log Medicaid spending and the predictive value of pretreatment trends among control observations. Additional methodological details are provided in the Supplementary Methods.

**Table 1.** Characteristics of students by Universal Free Meals adoption cohort.

	2016	2017	2018	2019
Students, *n*	85 583	82 249	76 586	71 730
UFM exposure, *n* (%)	4368 (5.1)	5006 (6.1)	3263 (4.3)	2600 (3.6)
Race/ethnicity, *n* (%)				
White	50 554 (59.1)	49 202 (59.8)	46 037 (60.1)	44 284 (61.7)
Black	17 676 (20.7)	15 942 (19.4)	14 121 (18.4)	11 074 (15.4)
Hispanic	13 209 (15.4)	12 850 (15.6)	12 227 (16.0)	12 136 (16.9)
Other	4144 (4.8)	4255 (5.2)	4201 (5.5)	4236 (5.9)
Female, *n* (%)	41 070 (48.0)	39 344 (47.8)	36 599 (47.8)	34 363 (47.9)
Age, mean (SD)	10.5 (3.1)	10.5 (3.1)	10.5 (3.1)	10.6 (3.1)
Childhood Opportunity Index, *n* (%)^a^				
Very low	17 667 (20.6)	15 703 (19.1)	13 369 (17.5)	10 959 (15.3)
Low	20 316 (23.7)	19 356 (23.5)	18 277 (23.9)	16 454 (22.9)
Moderate	20 821 (24.3)	19 969 (24.3)	18 990 (24.8)	18 257 (25.5)
High	17 424 (20.4)	17 636 (21.4)	16 374 (21.4)	16 286 (22.7)
Very high	9355 (10.9)	9585 (11.7)	9576 (12.5)	9774 (13.6)
PASSE participation, *n* (%)b^b^	0 (0.0)	0 (0.0)	9659 (12.6)	10 817 (15.1)
Annual Medicaid spending by service category, mean (SD)^c^				
Inpatient	704 (6024)	685 (6253)	711 (8332)	696 (6625)
Emergency Dept	73 (161)	73 (158)	71 (156)	72 (162)
Outpatient	1830 (5328)	1822 (4914)	1759 (4832)	1646 (4457)
Pharmacy	815 (16 341)	850 (16 909)	812 (6206)	812 (7314)
Total	3422 (19 063)	3430 (19 478)	3354 (12 778)	3227 (11 955)

Source: Authors’ analysis of linked Arkansas Department of Education records and Arkansas All-Payer Claims Database, 2013-2020. Notes: Counts represent all treated and control students contributing observations to each adoption cohort. The adoption cohorts comprise the sub-experiments used in the stacked difference-in-differences design.

^a^Childhood Opportunity Index categories reflect the value observed in the first year of each student's sub-experiment event window.

^b^PASSE participation indicates enrollment in the Provider-Led Arkansas Shared Savings Entity program during any year of the 4-year event window.

^c^Spending measures represent mean annual Medicaid expenditures averaged across the 2 prepolicy years (event times −2 and −1) for each adoption cohort.

We estimated the effect of UFM on log Medicaid expenditures using a DiD framework with sub-experiment-specific fixed effects for individual and event time:


$$\log\;(Spending_{ist})=\beta_{0}+\beta_{1}\mathrm{UF}M_{ist}+\alpha_{is}+\lambda_{st}+\varepsilon_{ist}$$

where $Spending_{ist}$ denotes annual Medicaid spending for individual *i* in sub-experiment *s* at event time *t*. The term $\alpha_{is}$ denotes individual-by-sub-experiment fixed effects, $\lambda_{st}$ denotes sub-experiment-specific event-time fixed effects, and $\mathrm{UF}M_{ist}$ is an indicator for exposure to UFM. The coefficient $\beta_{1}$ has the interpretation as the ATT. Because 92.5% of student-year observations had nonzero expenditures, we modeled log annual Medicaid spending, setting zero and very small values (below one dollar) to $1 prior to transformation. With a log-transformed outcome, coefficients represent relative changes in expected spending. Individual-level fixed effects absorb time-invariant differences across individuals within a given adoption cohort and common shocks at each relative event time. We also estimated an event-study specification replacing the single treatment indicator with event-time indicators interacted with treatment status, allowing formal assessment of the parallel trends assumption and characterization of dynamic effects in post-adoption years.

For all models, standard errors were clustered at both the school and student levels. As a robustness check, we estimated an alternative stacked specification using common event-time indicators and a treatment indicator rather than individual-by-sub-experiment fixed effects.

### Analysis of spending categories

To provide additional context for the overall ATT, we classified spending into mutually exclusive service categories and clinical groupings and estimated policy associations separately for each. Claims were classified as inpatient, emergency department, outpatient, or pharmacy spending using APCD documentation and prior claims-classification guidance.^35,39^ Within each service category, spending was further classified into mental, behavioral, and neurodevelopmental disorders (MBD), respiratory diseases (RSP), and all other spending using ICD-10-CM codes mapped to the Agency for Healthcare Research and Quality (AHRQ). Clinical Classifications Software Refined (CCSR) categories (ICD-9-CM codes from claims prior to October 2015 were first translated to ICD-10-CM using the AHRQ crosswalk).^40^ For pharmacy claims, we constructed analogous categories using the first 2 digits of the Generic Product Identifier codes, mapping psychotherapeutic and neurologic agents to MBD, respiratory agents and antihistamines to RSP, and all remaining medications to an “other” category.

Within the broad MBD category, we conducted follow-up analyses using more granular CCSR diagnostic categories and pharmacy therapeutic classes to identify the conditions and medication groups accounting for the largest shares of spending. Within the ADHD, antinarcolepsy, antiobesity, and anorexiants therapeutic class, medications commonly prescribed for ADHD were identified using generic and brand medication names.^41^ We also examined the distribution of spending across participants and nonparticipants in the PASSE program to better characterize spending patterns among children with complex behavioral health needs. These descriptive analyses were conducted using the underlying claims data rather than the stacked DiD sample. Consequently, spending totals are not directly comparable to those from the stacked DiD analyses, in which observations may contribute to multiple sub-experiments.

We next estimated policy associations for each spending category using generalized linear mixed models with a Tweedie distribution and log link, including random intercepts for sub-experiment-specific individual identifiers and adjusting for race/ethnicity, gender, age, and neighborhood opportunity as measured by the Childhood Opportunity Index (COI) version 2.0. COI is a census-tract-level composite measure of neighborhood opportunity across education, health/environmental, and social/economic domains, typically grouped into 5 levels.^42^ These models applied the same stacked DiD data structure described above (Methods). Unlike total annual Medicaid spending, category-specific outcomes contained a large proportion of zero values because many children had no spending within a given service or clinical category during a given year.

The Tweedie distribution accommodates the semicontinuous nature of such expenditures, which exhibit a mass at zero and right-skewed positive values while retaining all observations in the analysis. This approach is well established for modeling healthcare spending with excess zeros and skewness.^43-47^ Nevertheless, the Tweedie models were to provide additional context for findings from the primary DiD findings on overall spending. GLM estimation was not used for the primary analysis because of stronger assumptions about the independence of the random effects. GLM can also be problematic in a DiD context.^48-50^

Tweedie models were estimated for pooled post-adoption and event-study specifications and provided marginal mean spending ratios, which represent relative differences in expected spending on the original dollar scale. Pooled post-adoption effects were estimated as the ratio of model-adjusted marginal mean expenditures between treated and control units, averaging over the observed covariate distribution. Event-study estimates were obtained by exponentiating the event-time interaction coefficients and their confidence limits, yielding dynamic spending ratios for each event time relative to the preadoption reference year.

All analyses were conducted using R statistical software (version 4.5.2).

### Limitations

Our study has several limitations. Assignment to UFM adoption was not random, and treated and control observations differ on observable characteristics. Although the stacked design, weighting strategy, and event-study diagnostics mitigate concerns related to staggered adoption and differential pretrends, unobserved time-varying factors coincident with UFM adoption may still influence estimates. While school-level eligibility requirements for the CEP or Provision 2 did not change over the study period, schools adopting UFM in later cohorts generally served more socioeconomically advantaged student populations than earlier adopters, reflecting differences in the timing of UFM adoption across schools. Consequently, the reported effects represent averages across adoption cohorts that differed in baseline socioeconomic characteristics and may mask heterogeneity in the effects of UFM.

Findings may not generalize to children with private insurance or attending private schools. Moreover, this study examined a single state. Arkansas's relatively high rates of child food insecurity and Medicaid enrollment may limit applicability to states with different demographic, economic, or healthcare contexts. The stacked DiD design included only 2 pre- and 2 post-adoption years per cohort, limiting our ability to assess pretreatment trends further in advance of adoption or to evaluate longer-term associations between UFM and healthcare expenditures.

## Study results

### Sample characteristics

The stacked DiD sample included 131 851 unique students contributing 1 264 592 student-year observations across 4 adoption cohorts (Table 1; Table A1). Of 1183 Arkansas public schools, 300 adopted UFM during the study period, with approximately 96% of first adoptions in the 4 cohorts occurring through the CEP and 4% through Provision 2.

Student characteristics were similar across cohorts. Approximately 59%-62% of students were White, 15%-21% Black, and 15%-17% Hispanic, with a balanced gender distribution (48% female) and a mean age of 10.5 years. Average annual total Medicaid spending ranged from $3187 to $3509 per student, with outpatient services accounting for the largest share of expenditures.

### Overall medicaid spending

**Figure 2.**
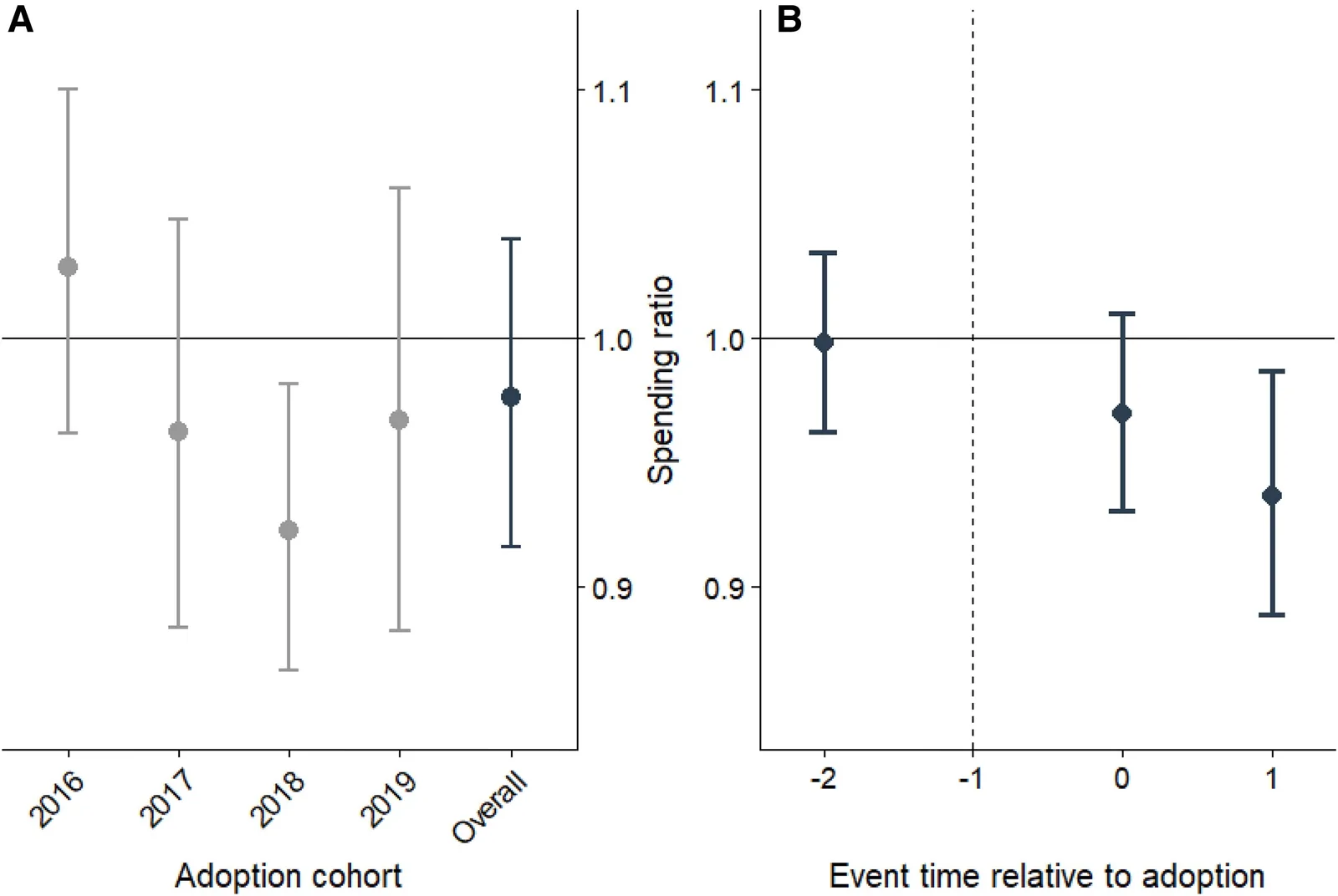
Estimated association between Universal Free Meals adoption and annual medicaid spending. Source: Authors’ analysis of linked Arkansas Department of Education records and Arkansas All-Payer Claims Database, 2013-2020. Notes: Panel A presents overall spending ratios estimated separately for each adoption cohort. The adoption cohorts comprise the individual sub-experiments in the stacked difference-in-differences design. The “Overall” estimate corresponds to the pooled stacked difference-in-differences model reported in the main text. Panel B presents event-study estimates from the pooled weighted stacked difference-in-differences model, with spending ratios expressed relative to the reference period (event time −1). Error bars indicate 95% confidence intervals. All models include individual-by-sub-experiment and sub-experiment-specific event-time fixed effects, with standard errors clustered at the school and student levels.

In the primary stacked DiD model with sub-experiment-specific fixed effects for individual and event time, UFM exposure corresponded to a spending ratio of 0.973 (95% confidence interval: 0.932 to 1.016) on total Medicaid spending (Figure 2). This estimate reflects within-student changes over time and accounts for staggered policy adoption across schools.

### Dynamic effects over time

Event-study estimates indicated no evidence of differential pretreatment trends in total Medicaid spending between treated and control observations after matching (spending ratio = 0.998; 95% CI: 0.0.962-1.035; Figure 2). In the first year after adoption, estimated changes in total Medicaid spending trended nonsignificantly lower (spending ratio = 0.970; 95% CI: 0.391-1.010). Total Medicaid spending declined significantly in the second adoption year (spending ratio = 0.937; 95% CI: 0.889-0.987), equivalent to a 6.3% reduction in total Medicaid spending.

### Medicaid spending by service type and clinical category

**Figure 3.**
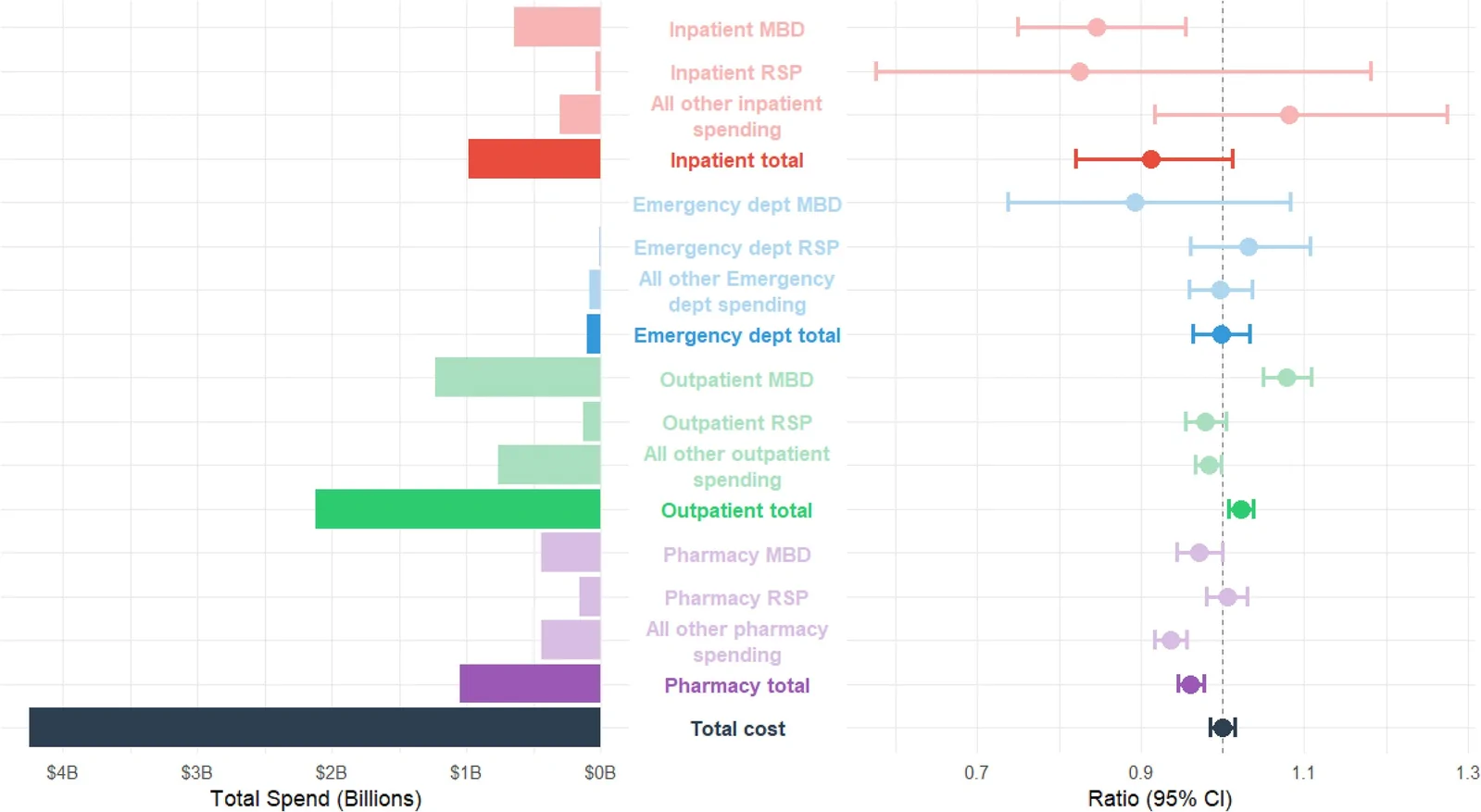
Changes in Medicaid spending by service type and clinical category following Universal Free Meals adoption. Source: Authors’ analysis of linked Arkansas Department of Education records and Arkansas All-Payer Claims Database, 2013-2020. Notes: The left panel displays total Medicaid spending by service type and clinical category, expressed in billions of dollars. The right panel displays spending ratios and 95% confidence intervals from weighted stacked difference-in-differences models estimating the effect of Universal Free Meals (UFM) on category-specific Medicaid expenditures. Estimates are derived from generalized linear mixed models with a Tweedie distribution and log link, adjusting for race/ethnicity, gender, age, and neighborhood opportunity (COI). Clinical categories include mental, behavioral, and neurodevelopmental disorders (MBD), respiratory conditions (RSP), and all other conditions.

Estimated associations from the Tweedie models provide additional context to the primary finding above. Adoption of UFM was not significantly associated with inpatient, outpatient, or pharmacy costs to manage or treat RSPs and ED costs (Figure 3). UFM adoption was associated with reductions in mental, behavioral, and neurodevelopmental disorders (MBD) in the inpatient setting (marginal effects ratio = 0.846; 95% CI: 0.750 to 0.954) and pharmacy MBD spending (marginal effects ratio = 0.971; 95% CI: 0.944 to 0.999). Pharmacy spending in categories other than MBD and respiratory conditions also declined significantly (marginal effects ratio = 0.937; 95% CI: 0.917 to 0.956). In contrast, outpatient MBD spending rose by approximately 8.0% (marginal effects ratio = 1.078; 95% CI: 1.049 to 1.108), contributing to an overall increase in total outpatient spending (marginal effects ratio = 1.022; 95% CI: 1.007 to 1.037) after UFM adoption. At the same time, outpatient spending for non-MBD conditions declined modestly (marginal effects ratio = 0.983; 95% CI: 0.967 to 0.998).

Supplementary event-study analyses from the Tweedie models produced temporal patterns consistent with the pooled estimates and confirmed the delayed reduction in overall Medicaid spending observed in the primary analysis (Table A2). Taken together, these findings indicate that shifts in Medicaid expenditures following UFM adoption were driven primarily by reductions in MBD-related inpatient and pharmacy spending, accompanied by increased outpatient MBD care.

Descriptive analyses indicated that spending was concentrated in a relatively small number of MBD categories, with mood disorders, depressive disorders, disruptive/impulse-control and conduct disorders, trauma- and stressor-related disorders, and neurodevelopmental disorders accounting for the largest shares of inpatient and outpatient MBD expenditures (Figure A1 and Table A3). PASSE participants accounted for a substantial proportion of MBD spending, representing 60.5% of inpatient MBD expenditures and 51.9% of outpatient MBD expenditures. MBD pharmacy spending was highly concentrated in the ADHD, antinarcolepsy, antiobesity, and anorexiants therapeutic class, which accounted for approximately 88.1% of total MBD pharmacy expenditures (Figure A2). ADHD medications accounted for 98.8% of spending within this therapeutic class and 87.0% of total MBD pharmacy spending. PASSE participants accounted for 36.9% of total MBD pharmacy spending and 36.4% of ADHD medication spending.

### Sensitivity analysis

Results were generally consistent across alternative specifications (Table A4), including a more parsimonious stacked DiD specification with common event-time indicators, relaxing the exclusive-Medicaid-payer requirement, excluding PASSE participants, and excluding the 2018-2019 cohort (whose final post-adoption year overlapped with COVID-19 onset). Excluding PASSE participants produced larger estimated reductions. Dynamic estimates remained negative across specifications, though significance varied for some event-time coefficients.

## Discussion

We examined whether UFM adoption in Arkansas public schools changed Medicaid expenditures among continuously enrolled children, using a stacked DiD design that accounts for staggered adoption and balances treated and control observations on pretreatment spending trends.

We found a significant reduction in Medicaid spending in the second year following UFM adoption but not over the first 2 postadoption years combined. These delayed reductions were concentrated in inpatient and pharmacy spending related to mental, behavioral, and neurodevelopmental disorders (MBD) and were accompanied by increases in outpatient MBD care, indicating meaningful shifts in patterns of healthcare utilization.

The MBD categories contributing to inpatient and outpatient MBD spending were largely overlapping, but inpatient spending was more heavily weighted toward mood disorders and depressive disorders, while outpatient spending was more heavily weighted toward disruptive disorders and neurodevelopmental disorders. PASSE participants accounted for approximately 61% of inpatient MBD spending, reflecting the substantial inpatient utilization among children with complex behavioral health needs. Pharmacy MBD spending was highly concentrated in ADHD medications, which accounted for approximately 87% of total MBD pharmacy expenditures. Together, these findings suggest that the observed reductions in inpatient MBD spending may have been associated with fewer or less severe mood and depressive disorder presentations requiring inpatient care.

The observed reallocation of MBD-related care is broadly consistent with the literature linking food insecurity and poor nutrition to children's mental and behavioral health.^25,51-54^ A growing literature documents associations between food insecurity and severity of ADHD symptoms, depression, and anxiety,^55-57^ as well as increased use of psychotropic medications.^53,58,59^ Food insecurity may also function as a chronic stressor that disrupts family functioning and the management of children's behavioral health needs.^60-62^ In addition, UFM increases participation in school meals that are required to meet federal nutrition standards, and higher dietary quality has been associated with improved emotional and behavioral functioning and lower levels of depressive and anxiety symptoms among children and adolescents.^63-66^ Prior studies have also shown that universal or expanded school meal programs are associated with improvements in student behavior, including reductions in disciplinary incidents, suspensions, and classroom disruptions.^1,67-70^ Together, these findings suggest plausible pathways by which improved nutrition and reduced food-related stress from UFM could reduce mental and behavioral health needs and inpatient spending as our findings suggest.

Nevertheless, administrative claims data reflect billed and paid services rather than symptom severity or clinical functioning; observed spending changes may capture shifts in prescribing or access rather than true changes in health status. Declines in higher-intensity services alongside increased outpatient MBD care may reflect changes in care delivery rather than reductions in the prevalence or severity of mental and behavioral health conditions. Accordingly, these findings should be viewed as hypothesis-generating rather than definitive, and replication integrating claims data with validated clinical and food security measures will be essential for clarifying mechanisms and heterogeneity across populations and school contexts.

The estimated reduction in total Medicaid spending in the second year following UFM adoption was modest but meaningful given Arkansas's overall Medicaid budget, which exceeds $9 billion annually. In our raw claims data, annual Medicaid spending among children exclusively covered by Medicaid in schools that had not yet adopted UFM averaged approximately $480 million before continuous-enrollment restrictions; applying the estimated 6.3% second-year reduction to this baseline would correspond to annual savings on the order of $30 million. Our findings suggest that the relevance of school meal policy, which is customarily evaluated through an educational and nutritional lens, may extend beyond schools and households to the healthcare system, particularly for publicly insured children.

## Conclusion

Significant reductions in Medicaid spending emerged in the second year following UFM adoption. This reduction was concentrated in inpatient and pharmacy spending related to mental, behavioral, and neurodevelopmental health services.

Exhibit list

## Supplementary Material

qxag196_Supplementary_Data

## References

[1] Spill MK, Trivedi R, Thoerig RC, et al Universal free school meals and school and student outcomes: a systematic review. JAMA Netw Open. 2024;7(8):e2424082. 10.1001/jamanetworkopen.2024.24082 39120904 PMC11316229

[2] Cohen JFW, Hecht AA, McLoughlin GM, Turner L, Schwartz MB. Universal school meals and associations with student participation, attendance, academic performance, diet quality, food security, and body mass index: a systematic review. Nutrients. 2021;13(3):911. 10.3390/nu13030911 33799780 PMC8000006

[3] Community eligibility provision . United States Department of Agriculture. Accessed October 28, 2025. https://web.archive.org/web/20260731214044/https://www.fna.usda.gov/cn/cep.

[4] Baker J . Community eligibility reduces hunger and decreases administrative burden for schools, report finds. Food Research & Action Center. Accessed April 21, 2025. https://frac.org/news/cepreportdec2024.

[5] Community eligibility: The key to hunger-free schools . Food Research & Action Center. Accessed July 10, 2026. https://frac.org/wp-content/uploads/CEP-Report-2024.pdf.

[6] Bylander A; Analyst SCNP. States show us what is possible with free healthy school meals for all policies. Accessed July 10, 2026. https://frac.org/blog/free-healthy-school-meals-for-all-policies.

[7] Food and Nutrition Administration . NSLP provisions 1, 2, and 3. United States Department of Agriculture. Accessed June 17, 2026. https://web.archive.org/web/20260804213838/https://www.fna.usda.gov/cn/provisions-1-2-and-3.

[8] Crepinsek MK, Singh A, Bernstein LS, McLaughlin JE. Dietary effects of universal-free school breakfast: findings from the evaluation of the school breakfast program pilot project. J Am Diet Assoc. 2006;106(11):1796–1803. 10.1016/j.jada.2006.08.013 17081831

[9] Leos-Urbel J, Schwartz AE, Weinstein M, Corcoran S. Not just for poor kids: the impact of universal free school breakfast on meal participation and student outcomes. Econ Educ Rev. 2013;36:88–107. 10.1016/j.econedurev.2013.06.007 24465073 PMC3900011

[10] Logan CW, Connor P, Harvill EL, et al Community Eligibility Provision evaluation. Nutrition Assistance Program report. United States Department of Agriculture. Accessed July 10, 2026. https://files.eric.ed.gov/fulltext/ED557961.pdf.

[11] Pokorney PE, Chandran A, Long MW. Impact of the community eligibility provision on meal counts and participation in Pennsylvania and Maryland national school lunch programs. Public Health Nutr. 2019;22(17):3281–3287. 10.1017/S1368980019002246 31409436 PMC10260558

[12] Ribar DC, Haldeman LA. Changes in meal participation, attendance, and test scores associated with the availability of universal free school breakfasts. Soc Serv Rev. 2013;87(2):354–385. 10.1086/671013

[13] Schwartz AE, Rothbart MW. Let them eat lunch: the impact of universal free meals on student performance. J Policy Anal Manag. 2020;39(2):376–410. 10.1002/pam.22175

[14] Tan ML, Laraia B, Madsen KA, Johnson RC, Ritchie L. Community eligibility Provision and school meal participation among student subgroups. J Sch Health. 2020;90(10):802–811. 10.1111/josh.12942 32754916

[15] Turner L, Guthrie JF, Ralston K. Community eligibility and other provisions for universal free meals at school: impact on student breakfast and lunch participation in California public schools. Transl Behav Med. 2019;9(5):931–941. 10.1093/tbm/ibz090 31328770

[16] Gordanier J, Ozturk O, Williams B, Zhan C. Free lunch for all! The effect of the Community Eligibility Provision on academic outcomes. Econ Educ Rev. 2020;77:101999. 10.1016/j.econedurev.2020.101999

[17] Schneider KR, Oslund J, Liu T. Impact of the Community Eligibility Provision program on school meal participation in Texas. Public Health Nutr. 2021;24(18):6534–6542. 10.1017/S1368980021003712 34462037 PMC11148575

[18] Buscemi J, Odoms-Young A, Yaroch AL, Hayman LL, Robertson TP, Fitzgibbon ML. Society of Behavioral Medicine (SBM) position statement: SBM supports retaining healthy school lunch policies. Transl Behav Med. 2015;5(3):357–359. 10.1007/s13142-015-0318-z 26327942 PMC4537455

[19] Cohen J, Schwartz MB. Documented success and future potential of the Healthy, Hunger-Free Kids Act. J Acad Nutr Diet. 2020;120(3):359–362. 10.1016/j.jand.2019.10.021 31948796 PMC7216560

[20] Adams EL, Raynor HA, Thornton LM, Mazzeo SE, Bean MK. Using digital imagery to quantify students’ added sugar intake at lunch in Title I schools with universal free meals. Prev Med Rep. 2020;20:101253. 10.1016/j.pmedr.2020.101253 33318885 PMC7723792

[21] Kleinman RE, Hall S, Green H, et al Diet, breakfast, and academic performance in children. Ann Nutr Metab. 2002;46 Suppl 1:24–30. 10.1159/000066399 PMC3275817 12428078

[22] Gross SM, Kelley TL, Augustyn M, Wilson MJ, Bassarab K, Palmer A. Household food security status of families with children attending schools that participate in the Community Eligibility Provision (CEP) and those with children attending schools that are CEP-eligible, but not participating. J Hunger Environ Nutr. 2021;16(2):281–296. 10.1080/19320248.2019.1679318

[23] Khan S, Pinckney RG, Keeney D, Frankowski B, Carney JK. Prevalence of food insecurity and utilization of food assistance program: an exploratory survey of a Vermont middle school. J Sch Health. 2011;81(1):15–20. 10.1111/j.1746-1561.2010.00552.x 21158861

[24] Poblacion A, Cook J, de Cuba S E, et al Can food insecurity be reduced in the United States by improving SNAP, WIC, and the Community Eligibility Provision? World Med Health Policy. 2017;9(4):435–455. 10.1002/wmh3.248

[25] Thomas MMC, Miller DP, Morrissey TW. Food insecurity and child health. Pediatrics. 2019;144(4):e20190397. 10.1542/peds.2019-0397 31501236

[26] Hatsu IE, Eiterman L, Stern M, et al Household food insecurity is associated with symptoms of emotional dysregulation in children with attention deficit hyperactivity disorder: the MADDY study. Nutrients. 2022;14(6):1306. 10.3390/nu14061306 35334963 PMC8952815

[27] Hill NE, Palakshappa D, Chua K-P. Chronic conditions and food insecurity in US children. JAMA Netw Open. 2025;8(9):e2533953. 10.1001/jamanetworkopen.2025.33953 41004147 PMC12475946

[28] Lu S, Perez L, Leslein A, Hatsu I. The relationship between food insecurity and symptoms of attention-deficit hyperactivity disorder in children: a summary of the literature. Nutrients. 2019;11(3):659. 10.3390/nu11030659 30893802 PMC6470829

[29] Green CD, Martinez AC, Becker SP. Examining food insecurity in relation to ADHD and cognitive disengagement syndrome symptoms in early adolescents. Res Child Adolesc Psychopathol. 2024;52(11):1649–1661. 10.1007/s10802-024-01226-5 38967900 PMC12376416

[30] Berkowitz SA, Basu S, Meigs JB, Seligman HK. Food insecurity and health care expenditures in the United States, 2011–2013. Health Serv Res. 2017;53(3):1600–1620. 10.1111/1475-6773.12730 28608473 PMC5980147

[31] Ratcliffe C, McKernan S-M, Zhang S. How much does the Supplemental Nutrition Assistance Program reduce food insecurity? Am J Agric Econ. 2011;93(4):1082–1098. 10.1093/ajae/aar026 25197100 PMC4154696

[32] Berkowitz SA, Seligman HK, Rigdon J, Meigs JB, Basu S. Supplemental Nutrition Assistance Program (SNAP) participation and health care expenditures among low-income adults. JAMA Intern Med. 2017;177(11):1642–1649. 10.1001/jamainternmed.2017.4841 28973507 PMC5710268

[33] Berkowitz SA, Palakshappa D, Rigdon J, Seligman HK, Basu S. Supplemental Nutrition Assistance Program participation and health care use in older adults: a cohort study. Ann Intern Med. 2021;174(12):1674–1682. 10.7326/M21-1588 34662150 PMC8893035

[34] Wing C, Freedman SM, Hollingsworth A. Stacked difference-in-differences. NBER Working Paper 32054 2024. National Bureau of Economic Research. Cambridge, MA, January 2024. 10.3386/w32054

[35] Arkansas all-payer claims database . Arkansas Center for Health Improvement. Accessed July 10, 2026. https://web.archive.org/web/20260804213957/https://www.arkansasapcd.net/Home/.

[36] ELSI—Elementary and Secondary Information System . National Center for Education Statistics. Accessed July 10, 2026. https://nces.ed.gov/ccd/elsi/tablegenerator.aspx.

[37] PASSE—Provider-led Arkansas Shared Savings Entity . Arkansas Department of Human Services. Accessed July 10, 2026. https://humanservices.arkansas.gov/divisions-shared-services/medical-services/healthcare-programs/passe/.

[38] Iacus SM, King G, Porro G. Causal inference without balance checking: coarsened exact matching. Polit Anal. 2012;20(1):1–24. 10.1093/pan/mpr013

[39] Beilstein-Wedel E, Vanneman M. Categorizing claims in the consolidated data set (CDS) inpatient vs outpatient. Veterans Affairs. Accessed July 10, 2026. https://www.hsrd.research.va.gov/centers/core/accent/community_care/Categorizing-Inpatient-and-Outpatient-Records-in-CDS.pdf.

[40] Data innovations—ICD-10-CM/PCS resources: Tools . Agency for Healthcare Research and Quality. Accessed July 10, 2026. https://web.archive.org/web/20260804214053/https://hcupus.ahrq.gov/datainnovations/icd10_tools.jsp.

[41] ADHD medications approved by the US food and drug administration . Children and Adults with Attention-Deficit/Hyperactivity Disorder (CHADD). Accessed July 10, 2026. https://d393uh8gb46l22.cloudfront.net/wp-content/uploads/2020/02/ADHD-Medications-Approved-by-FDA-2.23.pdf.

[42] Noelke C, McArdle N, Baek M, et al Child opportunity index 2.0. Technical documentation. 2020. Accessed July 10, 2026. https://www.diversitydatakids.org/sites/default/files/2020-02/ddk_coi2.0_technical_documentation_20200212.pdf.

[43] Hao S . Modeling hospitalization medical expenditure of the elderly in China. Econ Anal Policy. 2023;79:450–461. 10.1016/j.eap.2023.06.020

[44] Ge Z . Modeling health expenditures using generalized linear models. 2024. Accessed July 10, 2026. https://conservancy.umn.edu/items/2a4fa237-15b4-4649-9e37-084cf460fc92.

[45] Liebert R, Sanders A, Kudel I. Modeling techniques for fitting healthcare resource use costs derived from patient reported counts of hospital, ER, and PCP visits. Value Health. 2017;20(9):A740. 10.1016/j.jval.2017.08.2041

[46] Smyth GK, Jørgensen B. Fitting Tweedie's compound poisson model to insurance claims data: dispersion modelling. ASTIN Bulletin: The Journal of the IAA. 2002;32(1):143–157. 10.2143/AST.32.1.1020

[47] Kurz CF . Tweedie distributions for fitting semicontinuous health care utilization cost data. BMC Med Res Methodol. 2017;17(1):171. 10.1186/s12874-017-0445-y 29258428 PMC5735804

[48] Cameron AC . Microeconometrics: Methods and Applications. Cambridge University; 2005.

[49] Angrist JD, Pischke J. Mostly Harmless Econometrics: An empiricist's companion. Princeton University Press; 2009.

[50] Cameron AC, Trivedi PK. Microeconometrics Using Stata, Volumes I and II. Stata Press; 2022.

[51] Knowles M, Rabinowich J, de Cuba S E, Cutts DB, Chilton M. “Do you wanna breathe or eat?”: parent perspectives on child health consequences of food insecurity, trade-offs, and toxic stress. Matern Child Health J. 2016;20(1):25–32. 10.1007/s10995-015-1797-8 26156827 PMC4712223

[52] Shankar P, Chung R, Frank DA. Association of food insecurity with children's behavioral, emotional, and academic outcomes: a systematic review. J Dev Behav Pediatr. 2017;38(2):135–150. 10.1097/DBP.0000000000000383 28134627

[53] Burke MP, Martini LH, Çayır E, Hartline-Grafton HL, Meade RL. Severity of household food insecurity is positively associated with mental disorders among children and adolescents in the United States. J Nutr. 2016;146(10):2019–2026. 10.3945/jn.116.232298 27581581

[54] Frongillo EA, Adebiyi VO, Boncyk M. Meta-review of child and adolescent experiences and consequences of food insecurity. Glob Food Secur. 2024;41:100767. 10.1016/j.gfs.2024.100767

[55] Zheng S, Ngo AL, Forman MR, et al Associations of household food insufficiency with childhood depression and anxiety: a nationwide cross-sectional study in the USA. BMJ Open. 2021;11(9):e054263. 10.1136/bmjopen-2021-054263 PMC8424875 34493526

[56] Whitaker RC, Phillips SM, Orzol SM. Food insecurity and the risks of depression and anxiety in mothers and behavior problems in their preschool-aged children. Pediatrics. 2006;118(3):e859–e868. 10.1542/peds.2006-0239 16950971

[57] Chen X, Yeung WJ. How food insecurity affects children's behavior problems in early childhood: the nutrition and family stress pathways. PLoS One. 2024;19(1):e0294109. 10.1371/journal.pone.0294109 38170704 PMC10763944

[58] Whittle HJ, Wolfe WR, Sheira LA, et al Associations between food insecurity and psychotropic medication use among women living with HIV in the United States. Epidemiol Psychiatr Sci. 2020;29:e113. 10.1017/S2045796020000232 32248873 PMC7214522

[59] Anderson KK, Clemens KK, Le B, et al Household food insecurity and health service use for mental and substance use disorders among children and adolescents in Ontario, Canada. CMAJ. 2023;195(28):E948–E955. 10.1503/cmaj.230332 37487614 PMC10365855

[60] Abraham S, Breeze P, Lambie-Mumford H, Sutton A. Household food insecurity and its impact on child and adolescent health outcomes in western high-income countries: a rapid review of mechanisms and associations. Public Health Nutr. 2025;29(1):e17. 10.1017/S1368980025101092 40968663 PMC12895477

[61] McLaughlin KA, Green JG, Alegría M, et al Food insecurity and mental disorders in a national sample of US adolescents. J Am Acad Child Adolesc Psychiatry. 2012;51(12):1293–1303. 10.1016/j.jaac.2012.09.009 23200286 PMC3632292

[62] Dean G, Vitolins MZ, Skelton JA, Ip EH, Lucas CB, Brown CL. The association of food insecurity with mental health in preschool-aged children and their parents. Pediatr Res. 2023;94(1):290–295. 10.1038/s41390-022-02458-1 36599944 PMC10318115

[63] O’neil A, Quirk SE, Housden S, et al Relationship between diet and mental health in children and adolescents: a systematic review. Am J Public Health. 2014;104(10):e31–e42. 10.2105/AJPH.2014.302110 PMC4167107 25208008

[64] Khalid S, Williams CM, Reynolds SA. Is there an association between diet and depression in children and adolescents? A systematic review. Br J Nutr. 2016;116(12):2097–2108. 10.1017/S0007114516004359 28093091

[65] Grajek M, Krupa-Kotara K, Białek-Dratwa A, et al Nutrition and mental health: a review of current knowledge about the impact of diet on mental health. Front Nutr. 2022;9:943998. 10.3389/fnut.2022.943998 36071944 PMC9441951

[66] Firth J, Gangwisch JE, Borisini A, Wootton RE, Mayer EA. Food and mood: how do diet and nutrition affect mental wellbeing? BMJ. 2020;369:m2382. 10.1136/bmj.m2382 32601102 PMC7322666

[67] Hecht AA, Pollack Porter KM, Turner L. Impact of the Community Eligibility Provision of the Healthy, Hunger-Free Kids Act on student nutrition, behavior, and academic outcomes: 2011–2019. Am J Public Health. 2020;110(9):1405–1410. 10.2105/AJPH.2020.305743 32584590 PMC7427252

[68] Kho A . Three Essays on School Reform. Vanderbilt University; 2018.

[69] Gordon NE, Ruffini KJ. School nutrition and student discipline: Effects of schoolwide free meals. National Bureau of Economic Research. 2018. Accessed July 10, 2026. https://www.nber.org/system/files/working_papers/w24986/w24986.pdf.

[70] Gordon N, Ruffini K. Schoolwide free meals and student discipline: effects of the Community Eligibility Provision. Educ Finance Policy. 2021;16(3):418–442. 10.1162/edfp_a_00307

