# Supplementary material for: Universal free school meals and Medicaid spending: evidence from linked education and claims data in Arkansas
Source: Health Aff Sch. 2026 Aug 5;4(8):qxag196. doi: 10.1093/haschl/qxag196 (PMC13489158; doi:10.1093/haschl/qxag196)
Supplement: Supplementary file 1 [file qxag196_supplementary_data.zip › revisedSupplement.docx]

**Supplementary Methods**

This section provides additional details on the stacked difference-in-differences design, matching procedure, and category-specific spending models used to estimate policy effects.

*Stacked Difference-Differences Design*

Recent work has highlighted the limitations of traditional two-way fixed effects difference-in-differences (DiD) estimators in settings with staggered treatment adoption and heterogeneous treatment effects. Several alternative approaches have been proposed, including decompositions of two-way fixed effects estimates,^37^ group-time average treatment effect estimators,^38^ and event-study methods that explicitly account for treatment timing.^39^ Guided by the literature and the structure of our data, we adopted the stacked sub-experiment design, which estimates treatment effects within balanced event windows while avoiding problematic comparisons between already-treated and newly-treated units. This approach was particularly well-suited to our setting because it allowed assessment of pre-treatment trends and dynamic post-treatment effects without requiring continuous observation over the full seven years of the study period.

*Matching and Weight Construction*

To strengthen the validity of the identifying assumptions of the stacked DiD design, we applied a weighting procedure that balanced treated and control observations on both pre-treatment trends and adoption-cohort membership. We defined a pre-treatment trend measure as the change in log total Medicaid spending between event times −2 and −1 for each student. Using coarsened exact matching (CEM), we constructed weighting strata based on two variables: (1) sub-experiment membership, which identifies the adoption cohort to which a given observation belongs, and (2) the pre-treatment trend in log total Medicaid spending. Weighting was performed directly on the existing categorical sub-experiment variable, while the continuous pre-treatment trend measure was first coarsened into categories before inclusion in the weighting procedure. CEM was used solely to construct balancing weights; no matched pairs were created. Treated observations received unit weights, while control observations were reweighted within strata to match the distribution of treated observations. Because Medicaid spending is highly skewed and exhibits substantial persistence over time, including the pre-treatment trend in the weighting procedure improved comparability between treated and control observations with respect to spending trajectories prior to UFM adoption. In addition, including sub-experiment membership in the weighting procedure balanced the relative distribution of treated and control observations across adoption cohorts, consistent with the weighting framework described by Wing et al.^34^ The resulting estimator targets the average treatment effect on the treated (ATT) within the stacked sample.

*Assessment of Regression to the Mean*

Matching on pre-treatment outcomes or outcome trends in DiD designs can introduce bias through regression to the mean (RTM), particularly when matching selects units with extreme pre-period values that subsequently revert toward their population mean once the matching constraint is released.^40,41^ At the same time, prior work has demonstrated that combining DiD with matching on pre-treatment outcomes or trends can reduce bias when outcomes are serially correlated and when pre-treatment outcomes are predictive of future outcomes.^42,43^ We therefore examined the degree of serial correlation in log Medicaid spending and the predictive value of pre-treatment trends among control observations.

Medicaid spending exhibited substantial persistence over time within individuals. Across stacked sub-experiments, log annual Medicaid spending was strongly correlated with its one-year lag (correlation = 0.635). Pre-treatment spending trends were also predictive of subsequent outcomes among control observations: a one-unit increase in the change in log Medicaid spending between the two pre-adoption years was associated with a 0.100 increase in log spending in post-adoption periods (p < 0.001). These findings support the use of matching on pre-treatment spending trends to improve comparability between treated and control observations.

*Event-Study Specification*

To assess pre-treatment trends and estimate dynamic treatment effects in the primary analysis of total annual Medicaid spending, we estimated an event-study version of the stacked DiD model. This specification replaced the single treatment indicator for UFM with interactions between treatment status and event-time indicators:

$$\log\left( {Spending}_{ist} \right)=\beta_{0}+\sum_{\begin{aligned} h=-2 \\ h\neq-1 \end{aligned}}^{1} \beta_{h}\left[ D_{is}\times1\left( t=h \right) \right]+\alpha_{is}+\lambda_{st}+\varepsilon_{ist}$$

where ${Spending}_{ist}$ denotes annual Medicaid spending for individual $i$ in sub-experiment $s$ at event time $t$; $D_{is}$ denotes the treated-group indicator within sub-experiment; and $1\left( t=h \right)$ is an indicator for event time $h$. The term $a_{is}$ denotes individual-by-sub-experiment fixed effects and $\lambda_{st}$ denotes sub-experiment-specific event-time fixed effects. The event-study interaction coefficients $\beta_{h}$ represent treatment effects at event time $h$ relative to the omitted reference period ($t=-1$).

*Models for Category-Specific Spending*

For category-specific spending analyses, we estimated generalized linear mixed models with a Tweedie distribution and log link:

$$\log\left( E\left[ {Spending}_{ist} \right] \right)=\beta_{0}+\beta_{1}{UFM}_{ist}+\gamma D_{is}+\lambda_{st}+\boldsymbol{\theta}^{\boldsymbol{'}}\mathbf{X}_{\boldsymbol{ist}}+u_{is}$$

where ${Spending}_{ist}$ represents category-specific Medicaid spending for individual $i$ in sub-experiment $s$ at event time $t$; ${UFM}_{ist}$ indicates exposure to Universal Free Meals; $D_{is}$ denotes the treated-group indicator within sub-experiment; and $\lambda_{st}$ represents sub-experiment-specific event-time indicators. The vector $\mathbf{X}_{\boldsymbol{ist}}$ includes race/ethnicity, gender, age, and neighborhood opportunity measured using the Childhood Opportunity Index. The term $u_{is}$ is a random intercept for sub-experiment-specific individual identifiers. Random intercepts were used in place of the individual-by-sub-experiment fixed effects employed in the primary linear models because the generalized linear mixed models did not converge with the large number of fixed effects required by the stacked DiD design. The treated-group indicator $D_{is}$, which is absorbed in the fixed-effects specification, was therefore included explicitly in these models. Event-study versions of the category-specific spending models replaced the pooled treatment indicator with interactions between treatment status and event-time indicators:

$$\log\left( E\left[ {Spending}_{ist} \right] \right)=\beta_{0}+\gamma D_{is}+\sum_{\begin{aligned} h=-2 \\ h\neq-1 \end{aligned}}^{1} \beta_{h}\left[ D_{is}\times1\left( t=h \right) \right]+\lambda_{st}+\boldsymbol{\theta}^{\boldsymbol{'}}\mathbf{X}_{\boldsymbol{ist}}+u_{is}$$

where the coefficients $\beta_{h}$ represent spending ratios comparing treated to control observations at event time $h$ relative to the omitted reference period ($t=-1$). Models were estimated on the original dollar scale using a log link. For pooled post-adoption analyses, spending ratios were estimated as the ratio of model-adjusted marginal mean expenditures between treated and control observations, averaging over the observed covariate distribution. For event-study analyses (Supplement Table A2), spending ratios were obtained by exponentiating the treatment-by-event-time interaction coefficients $\beta_{h}$ and their confidence limits.

**Table A1.** Pre-policy characteristics of treated and comparison students by adoption cohort, before and after weighting

|  |  | **2016** | | | **2017** | | | **2018** | | | **2019** | | |
| --- | --- | --- | --- | --- | --- | --- | --- | --- | --- | --- | --- | --- | --- |
|  |  | **Treated** | **Comparison unweighted** | **Comparison weighted** | **Treated** | **Comparison unweighted** | **Comparison weighted** | **Treated** | **Comparison unweighted** | **Comparison weighted** | **Treated** | **Comparison unweighted** | **Comparison weighted** |
| **Students,** n | | 4368 | 81215 | 86262.3 | 5006 | 77243 | 98862 | 3263 | 73323 | 64440 | 2600 | 69130 | 51346.6 |
| **Race/ethnicity,** n (%) | | |  |  |  |  |  |  |  |  |  |  |  |
|  | White | 1598 (36.6) | 48956 (60.3) | 51935 (60.2) | 1985 (39.7) | 47217 (61.1) | 60391 (61.1) | 1475 (45.2) | 44562 (60.8) | 39033 (60.6) | 1513 (58.2) | 42771 (61.9) | 31762 (61.9) |
|  | Black | 2252 (51.6) | 15424 (19.0) | 16442 (19.1) | 2349 (46.9) | 13593 (17.6) | 17420 (17.6) | 1326 (40.6) | 12795 (17.5) | 11341 (17.6) | 816 (31.4) | 10258 (14.8) | 7629 (14.9) |
|  | Hispanic | 448  (10.3) | 12761 (15.7) | 13549 (15.7) | 501 (10.0) | 12349 (16.0) | 15821 (16.0) | 325 (10.0) | 11902 (16.2) | 10479 (16.3) | 192 (7.4) | 11944 (17.3) | 8865 (17.3) |
|  | Other | 70 (1.6) | 4074 (5.0) | 4337 (5.0) | 171 (3.4) | 4084 (5.3) | 5231 (5.3) | 137 (4.2) | 4064 (5.5) | 3588 (5.6) | 79 (3.0) | 4157 (6.0) | 3091 (6.0) |
| **Female,** n (%) | | 2117 (48.5) | 38953 (48.0) | 41326 (47.9) | 2399 (47.9) | 36945 (47.8) | 47262 (47.8) | 1561 (47.8) | 35038 (47.8) | 30760 (47.7) | 1245 (47.9) | 33118 (47.9) | 24585 (47.9) |
| **Age,** mean (SD) | | 9.6 (2.9) | 9.9 (2.9) | 9.9 (2.9) | 10.0 (2.8) | 9.9 (2.9) | 9.9 (2.9) | 9.5 (3.0) | 10.0 (2.9) | 10.0 (2.9) | 9.7 (2.9) | 10.0 (2.9) | 10.0 (2.9) |
| **Childhood Opportunity Index,** n (%) | | | |  |  |  |  |  |  |  |  |  |  |
|  | Very Low | 2507 (57.4) | 15490 (19.1) | 16482 (19.1) | 2479 (49.5) | 13746 (17.8) | 17606 (17.8) | 1391 (42.6) | 12395 (16.9) | 10951 (17.0) | 1021 (39.3) | 10317 (14.9) | 7662 (14.9) |
|  | Low | 1074 (24.6) | 19626 (24.2) | 20817.0 (24.1) | 1032 (20.6) | 18646 (24.1) | 23868 (24.1) | 779 (23.9) | 17851 (24.3) | 15682 (24.3) | 608 (23.4) | 16272 (23.5) | 12079 (23.5) |
|  | Moderate | 606 (13.9) | 19801 (24.4) | 21033 (24.4) | 944 (18.9) | 18716 (24.2) | 23943 (24.2) | 764 (23.4) | 18000 (24.5) | 15812 (24.5) | 508 (19.5) | 17409 (25.2) | 12941 (25.2) |
|  | High | 140 (3.2) | 17151 (21.1) | 18213 (21.1) | 467 (9.3) | 16846 (21.8) | 21557 (21.8) | 172 (5.3) | 15774 (21.5) | 13840 (21.5) | 412 (15.8) | 15596 (22.6) | 11590 (22.6) |
|  | Very High | 41 (0.9) | 9147 (11.3) | 9717 (11.3) | 84 (1.7) | 9289 (12.0) | 11887 (12.0) | 157 (4.8) | 9303 (12.7) | 8156 (12.7) | 51 (2.0) | 9536 (13.8) | 7075 (13.8) |
| **PASSE** n (%) | | 0 (0.0) | 0 (0.0) | 0.0 (0.0) | 0 (0.0) | 0 (0.0) | 0 (0.0) | 463 (14.2) | 9196 (12.5) | 7921 (12.3) | 565 (21.7) | 10252 (14.8) | 7539 (14.7) |
| **Annual Medicaid spending by service category**, mean (SD) | | | | | |  |  |  |  |  |  |  |  |
|  | Inpatient | 696 (6309) | 705 (6009) | 700 (6006) | 642 (5389) | 688 (6304) | 682 (6218) | 1033 (7117) | 697 (8381) | 706 (8440) | 1157 (8173) | 679 (6560) | 692 (6552) |
|  | Emergency Dept | 74 (140) | 73 (162) | 73 (162) | 74 (153) | 73 (158) | 73 (158) | 71 (138) | 71 (157) | 70 (156) | 80 (151) | 72 (162) | 72 (162) |
|  | Outpatient | 1613 (4319) | 1841 (5376) | 1827 (5357) | 1967 (3736) | 1813 (4980) | 1805 (4970) | 1854 (3955) | 1755 (4867) | 1721 (4794) | 1864 (3250) | 1638 (4496) | 1621 (4445) |
|  | Pharmacy | 632 (1864) | 825 (16769) | 818 (16706) | 755 (1976) | 856 (17442) | 853 (17398) | 934 (8334) | 807 (6094) | 785 (5985) | 652 (1524) | 818 (7444) | 803 (7341) |
|  | Total | 3015 (9115) | 3444 (19454) | 3417 (19388) | 3438 (7977) | 3430 (19996) | 3412 (19926) | 3893 (13165) | 3330 (12760) | 3282 (12698) | 3753 (9958) | 3208 (12023) | 3189 (11926) |

**Table A2.** Event-study estimates by service type and clinical category

| **Outcome** | **Pre-treatment (t = −2)** | **Year of adoption (t = 0)** | **One year after adoption (t = 1)** |
| --- | --- | --- | --- |
| **Inpatient** |  |  |  |
| MBD | 0.898 (0.753–1.070) | 0.824 (0.696–0.975) | 0.738 (0.623–0.874) |
| RSP | 0.993 (0.604–1.635) | 1.009 (0.600–1.697) | 0.932 (0.554–1.568) |
| All Other | 0.807 (0.634–1.027) | 1.036 (0.821–1.308) | 0.973 (0.774–1.224) |
| Total | 0.879 (0.755–1.023) | 0.889 (0.767–1.030) | 0.813 (0.702–0.942) |
| **Emergency Department** |  |  |  |
| MBD | 0.763 (0.556–1.045) | 0.865 (0.660–1.133) | 0.837 (0.646–1.085) |
| RSP | 1.017 (0.918–1.126) | 1.045 (0.942–1.159) | 1.100 (0.994–1.218) |
| All Other | 0.975 (0.921–1.031) | 1.009 (0.955–1.067) | 1.035 (0.979–1.093) |
| Total | 0.975 (0.926–1.025) | 1.011 (0.962–1.063) | 1.036 (0.986–1.088) |
| **Outpatient** |  |  |  |
| MBD | 0.998 (0.961–1.036) | 1.032 (0.993–1.072) | 1.013 (0.974–1.054) |
| RSP | 0.997 (0.963–1.032) | 0.959 (0.925–0.994) | 0.991 (0.956–1.027) |
| All Other | 1.012 (0.989–1.035) | 1.007 (0.985–1.030) | 1.010 (0.987–1.033) |
| Total | 1.001 (0.980–1.022) | 1.008 (0.987–1.029) | 0.996 (0.975–1.017) |
| **Pharmacy** |  |  |  |
| MBD | 1.051 (1.011–1.094) | 0.991 (0.952–1.032) | 0.973 (0.934–1.014) |
| RSP | 0.994 (0.960–1.029) | 0.987 (0.953–1.022) | 1.010 (0.975–1.047) |
| All Other | 0.989 (0.961–1.018) | 0.968 (0.940–0.997) | 0.940 (0.912–0.968) |
| Total | 1.011 (0.987–1.035) | 0.983 (0.960–1.007) | 0.961 (0.938–0.985) |
| **Overall Medicaid Spending** | 0.989 (0.969–1.010) | 0.992 (0.971–1.013) | 0.974 (0.953–0.995) |

**SOURCE** Authors’ analysis of linked Arkansas Department of Education records and Arkansas All-Payer Claims Database, 2013–2020. **NOTES** All estimates are spending ratios with 95% confidence intervals. Estimates were obtained from event-study specifications of the category-specific spending models. Category-specific spending outcomes were modeled using generalized linear mixed models with a Tweedie distribution and log link:

$$\log\left( E\left[ {Spending}_{ist} \right] \right)=\beta_{0}+\gamma D_{is}+\sum_{\begin{aligned} h=-2 \\ h\neq-1 \end{aligned}}^{1} \beta_{h}\left[ D_{is}\times1\left( t=h \right) \right]+\lambda_{st}+\boldsymbol{\theta}^{\boldsymbol{'}}\mathbf{X}_{\boldsymbol{ist}}+u_{is}$$

where ${Spending}_{ist}$ represents category-specific Medicaid spending for individual $i$ in sub-experiment $s$ at event time $t$; $D_{is}$ denotes the treated-group indicator within sub-experiment; $1\left( t=h \right)$ is an indicator for event time $h$; and $\lambda_{st}$ represents sub-experiment-specific event-time indicators. The vector $\mathbf{X}_{\boldsymbol{ist}}$ includes race/ethnicity, gender, age, and neighborhood opportunity measured using the Childhood Opportunity Index. The term $u_{is}$ is a random intercept for sub-experiment-specific individual identifiers. The event-study interaction coefficients $\beta_{h}$ represent spending ratios comparing treated to control observations relative to the omitted reference period ($t=-1$). Spending ratios and 95% confidence intervals were obtained by exponentiating the event-study interaction coefficients and their confidence limits.

**Table A3.** PASSE share of spending for major mental, behavioral, and neurodevelopmental disorder categories by service type

|  | **Category** | **Description** | **Total Spending ($M)** | **PASSE Spending ($M)** | **PASSE (%)** |
| --- | --- | --- | --- | --- | --- |
| **Inpatient** | |  |  |  |  |
|  | MBD004 | Other specified and unspecified mood disorders | 235.9 | 153.1 | 64.9 |
|  | MBD002 | Depressive disorders | 143.7 | 77.8 | 54.1 |
|  | MBD008 | Disruptive, impulse-control and conduct disorders | 65.9 | 37.4 | 56.8 |
|  | MBD007 | Trauma- and stressor-related disorders | 54.3 | 36.0 | 66.3 |
|  | MBD014 | Neurodevelopmental disorders | 46.3 | 25.6 | 55.2 |
|  | MBD012 | Suicidal ideation/attempt/intentional self-harm | 45.0 | 29.6 | 65.8 |
|  | MBD003 | Bipolar and related disorders | 33.8 | 17.6 | 52.0 |
|  | MBD001 | Schizophrenia spectrum and other psychotic disorders | 24.4 | 14.6 | 59.8 |
|  | MBD005 | Anxiety and fear-related disorders | 14.6 | 9.9 | 68.0 |
|  | MBD013 | Miscellaneous mental and behavioral disorders/conditions | 12.3 | 7.3 | 59.6 |
|  |  | All other MBD categories | 5.2 | 3.4 | 65.3 |
|  |  | Inpatient total | 681.5 | 412.3 | 60.5 |
| **Outpatient** | |  |  |  |  |
|  | MBD008 | Disruptive, impulse-control and conduct disorders | 248.2 | 141.2 | 56.9 |
|  | MBD014 | Neurodevelopmental disorders | 191.3 | 90.8 | 47.4 |
|  | MBD004 | Other specified and unspecified mood disorders | 151.7 | 89.5 | 59.0 |
|  | MBD007 | Trauma- and stressor-related disorders | 150.4 | 73.3 | 48.7 |
|  | MBD002 | Depressive disorders | 111.7 | 49.6 | 44.4 |
|  | MBD005 | Anxiety and fear-related disorders | 107.5 | 56.0 | 52.0 |
|  | MBD003 | Bipolar and related disorders | 21.5 | 11.3 | 52.8 |
|  | MBD001 | Schizophrenia spectrum and other psychotic disorders | 5.3 | 2.4 | 46.1 |
|  | MBD013 | Miscellaneous mental and behavioral disorders/conditions | 3.6 | 1.6 | 43.5 |
|  | MBD006 | Obsessive-compulsive and related disorders | 3.2 | 1.6 | 50.4 |
|  |  | All other MBD categories | 3.9 | 1.4 | 35.6 |
|  |  | Outpatient total | 998.3 | 518.6 | 51.9 |

**SOURCE** Authors’ analysis of linked Arkansas Department of Education records and Arkansas All-Payer Claims Database, 2013–2020. **NOTES** Mental and behavioral disorder (MBD) categories were identified using the Clinical Classifications Software Refined (CCSR) diagnostic classification system. Spending reflects total inpatient and outpatient expenditures aggregated across all children and study years. Descriptive summaries were calculated from the underlying claims data rather than the stacked DiD sample, therefore spending totals are not directly comparable to those reported in the primary analyses. PASSE spending represents expenditures incurred by children who were ever enrolled in a Provider-Led Arkansas Shared Savings Entity (PASSE) during the study period. Spending is reported in millions of dollars, and PASSE (%) represents the percentage of category-specific spending attributable to PASSE participants.

**Table A4.** Sensitivity analyses for the stacked difference-in-differences model

|  | **Primary stacked DiD model** | **Reduced FE specification** | **Additional payers included** | **PASSE participants excluded** | **COVID cohort excluded** |
| --- | --- | --- | --- | --- | --- |
| Aggregate post-period effect | 0.973 (0.932–1.016) | 0.976 (0.917–1.040) | 0.967 (0.928–1.007) | 0.949 (0.906–0.994) | 0.974 (0.928–1.022) |
| **Event-study estimates** |  |  |  |  |  |
| Pre-treatment (t = −2) | 0.998 (0.962–1.035) | 0.998 (0.960–1.037) | 0.995 (0.961–1.031) | 1.008 (0.968–1.049) | 0.997 (0.958–1.039) |
| Year of adoption (t = 0) | 0.970 (0.931–1.010) | 0.970 (0.918–1.025) | 0.967 (0.929–1.007) | 0.948 (0.907–0.992) | 0.978 (0.934–1.025) |
| One year after adoption (t = 1) | 0.937 (0.889–0.987) | 0.937 (0.871–1.006) | 0.927 (0.880–0.976) | 0.911 (0.860–0.966) | 0.948 (0.895–1.003) |
| Observations | 1,264,592 | 1,264,592 | 1,408,112 | 1,170,552 | 977,672 |

**SOURCE** Authors’ analysis of linked Arkansas Department of Education records and Arkansas All-Payer Claims Database, 2013–2020. **NOTES** All estimates are spending ratios with 95% confidence intervals. The primary model includes individual-by-sub-experiment and sub-experiment-specific event-time fixed effects:

$$\log\left( {Spending}_{ist} \right)=\beta_{0}+\beta_{1}\mathrm{UFM}_{ist}+\alpha_{is}+\lambda_{st}+\varepsilon_{ist}$$

where $i$ indexes individual, $s$ indexes sub-experiment, and $t$ indexes event time relative to policy adoption. The term $a_{is}$ denotes individual-by-sub-experiment fixed effects, $\lambda_{st}$ denotes sub-experiment-specific event-time fixed effects, and $\mathrm{UFM}_{ist}$ is an indicator for exposure to UFM. All specifications in Table A4 use this model except the “Reduced FE specification,” which replaces these with a treated-group indicator and a common set of event-time indicators across sub-experiments:

$$\log\left( {Spending}_{ist} \right)=\beta_{0}+\beta_{1}\mathrm{UFM}_{ist}+D_{is}+\lambda_{t}+\varepsilon_{ist}$$

where $D_{is}$ denotes the treated-group indicator within each sub-experiment and $\lambda_{t}$ represents event-time indicators that are common across sub-experiments. The "Additional payers included" specification relaxes the requirement that Medicaid be the exclusive payer while maintaining all other inclusion criteria and analytic steps. The "PASSE participants excluded" specification excludes children who ever participated in the Provider-Led Arkansas Shared Savings Entity (PASSE) program while maintaining all other inclusion criteria and analytic steps. The "Exclude COVID cohort" specification excludes the 2018–2019 adoption cohort, whose final post-adoption year overlapped with the onset of the COVID-19 pandemic, while maintaining all other inclusion criteria and analytic steps. Event-study estimates replace $\mathrm{UFM}_{ist}$ with interactions between treatment status and event-time indicators. All models apply analytic weights derived from coarsened exact matching and cluster standard errors at the school and student levels.

**Figure A1.** Total inpatient and outpatient spending for major mental, behavioral, and neurodevelopmental disorder categories

**SOURCE** Authors’ analysis of linked Arkansas Department of Education records and Arkansas All-Payer Claims Database, 2013–2020. **NOTES** Mental and behavioral disorder (MBD) categories were identified using the Clinical Classifications Software Refined (CCSR) diagnostic classification system. Bars display total inpatient and outpatient spending aggregated across all children and study years within each category. Descriptive summaries were calculated from the underlying claims data rather than the stacked DiD sample, therefore spending totals are not directly comparable to those reported in the primary analyses. Categories are ordered by total combined spending across inpatient and outpatient services. Spending is reported in millions of dollars.

**Figure A2.** Total pharmacy spending by mental, behavioral, and neurodevelopmental disorder therapeutic class

**SOURCE** Authors’ analysis of linked Arkansas Department of Education records and Arkansas All-Payer Claims Database, 2013–2020. **NOTES** Mental, behavioral, and neurodevelopmental disorder (MBD) pharmacy categories were defined using the first two digits of the Generic Product Identifier (GPI) therapeutic classification system. Spending reflects total pharmacy expenditures aggregated across all children and study years. Descriptive summaries were calculated from the underlying claims data rather than the stacked DiD sample, therefore spending totals are not directly comparable to those reported in the primary analyses. Categories are ordered by total spending. Spending is reported in millions of dollars.
